# Safety and immunogenicity of SARS-CoV-2 variant mRNA vaccine boosters in healthy adults: an interim analysis

**DOI:** 10.1038/s41591-021-01527-y

**Published:** 2021-09-15

**Authors:** Angela Choi, Matthew Koch, Kai Wu, Laurence Chu, LingZhi Ma, Anna Hill, Naveen Nunna, Wenmei Huang, Judy Oestreicher, Tonya Colpitts, Hamilton Bennett, Holly Legault, Yamuna Paila, Biliana Nestorova, Baoyu Ding, David Montefiori, Rolando Pajon, Jacqueline M. Miller, Brett Leav, Andrea Carfi, Roderick McPhee, Darin K. Edwards

**Affiliations:** 1 Moderna Inc., Cambridge, MA USA; 2 Benchmark Research, Austin, TX USA; 3 Immune Assay Team at Duke University Medical Center, Duke University, Durham, NC USA

**Keywords:** Viral infection, RNA vaccines

## Abstract

The emergence of SARS-CoV-2 variants of concern (VOCs) and variants of interest (VOIs) with decreased susceptibility to neutralization has generated interest in assessments of booster doses and variant-specific vaccines. Clinical trial participants who received a two-dose primary series of the COVID-19 vaccine mRNA-1273 approximately 6 months earlier entered an open-label phase 2a study (NCT04405076) to evaluate the primary objectives of safety and immunogenicity of a single booster dose of mRNA-1273 or variant-modified mRNAs, including multivalent mRNA-1273.211. As the trial is currently ongoing, this exploratory interim analysis includes preliminary descriptive results only of four booster groups (*n* = 20 per group). Immediately before the booster dose, neutralizing antibodies against wild-type D614G virus had waned (*P* < 0.0001) relative to peak titers against wild-type D614G measured 1 month after the primary series, and neutralization titers against B.1.351 (Beta), P.1 (Gamma) and B.1.617.2 (Delta) VOCs were either low or undetectable. Both the mRNA-1273 booster and variant-modified boosters were safe and well-tolerated. All boosters, including mRNA-1273, numerically increased neutralization titers against the wild-type D614G virus compared to peak titers against wild-type D614G measured 1 month after the primary series; significant increases were observed for mRNA-1273 and mRNA-1273.211 (*P* < 0.0001). In addition, all boosters increased neutralization titers against key VOCs and VOIs, including B.1.351, P.1. and B.1.617.2, that were statistically equivalent to peak titers measured after the primary vaccine series against wild-type D614G virus, with superior titers against some VOIs. This trial is ongoing.

## Abstract (web summary)

Preliminary and exploratory analyses show that a third dose of the COVID-19 vaccine mRNA-1273 or variant-modified boosters can boost levels of neutralizing antibodies against SARS-CoV-2 variants.

## Main

Several severe acute respiratory syndrome coronavirus 2 (SARS-CoV-2) vaccines targeting the viral spike (S) protein have been developed^1^. One such vaccine, mRNA-1273 (Moderna), a lipid nanoparticle-encapsulated mRNA vaccine encoding the S protein of the Wuhan-Hu-1 isolate with two proline mutations introduced to stabilize the S protein into the prefusion conformation, had an acceptable safety profile and induced anti-SARS-CoV-2 immune responses in phase 1 (NCT04283461)^2,3^ and phase 2 (NCT04405076)^4^ trials in adults. In the phase 3 Coronavirus Efficacy (COVE) trial (NCT04470427), mRNA-1273 provided 94% efficacy against symptomatic COVID-19 disease in more than 30,000 participants^5^. Subsequently, mRNA-1273 received emergency use authorization from several global regulatory bodies, including the U.S. Food and Drug Administration^6–8^.

Although SARS-CoV-2 vaccines, such as mRNA-1273, are highly effective in reducing detectable symptomatic infections and severe complications of Coronavirus Disease 2019 (COVID-19)^5^, several viral variants with changes in the S protein have emerged, some of which have been identified as VOCs (Alpha (B.1.1.7), Beta (B.1.351), Gamma (P.1) and Delta (B.1.617.2))^9–11^. Statistical models predict that protection from severe COVID-19 might be driven by neutralizing antibody levels considerably lower than the neutralizing responses elicited by mRNA vaccines^12^. Furthermore, a rapid anamnestic response might be generated upon subsequent exposure to VOCs by vaccination-induced germinal center memory B cells^13^. However, reduced efficacy against the B.1.351 (refs. ^14,15^) and B.1.617.2 (ref. ^16^) variants has been reported for some COVID-19 vaccines.

In a previous study, the neutralizing capacity of sera collected from participants 7 d after completion of the mRNA-1273 primary series against VOCs^17,18^ was assessed using a previously described research grade vesicular stomatitis virus (VSV)-based SARS-CoV-2 pseudovirus neutralization (PsVN) assay^19^. Neutralizing antibody titers measured in sera from eight mRNA-1273 phase 1 trial participants were reduced 2.1- to 8.4-fold against the B.1.617.2, P.1 and B.1.351 variants^17,18^. Currently, a neutralizing antibody titer threshold predictive of protection from SARS-CoV-2 infection in humans is unknown. However, the reduction of in vitro neutralizing antibody titers against variants relative to the wild-type D614G virus raises the possibility of breakthrough infections and waning efficacy for the current SARS-CoV-2 vaccines.

To address this potential risk, modified versions of the prototype mRNA-1273 vaccine that contain the genetic sequence of the variant S protein continue to be developed. These variant vaccines are designed to stimulate an immune response against key sites of neutralization that have been altered on the S protein of variant viruses and, in the case of a multivalent vaccine, simultaneously against the wild-type strain. Here we report data from an exploratory interim analysis of the preliminary safety and immunogenicity of single booster doses of mRNA-1273 (50 µg), modified mRNA-1273.351 (20 or 50 µg) encoding the S protein of B.1.351 and multivalent mRNA-1273.211 (a 1:1 mix of mRNA-1273 (25 µg) and mRNA-1273.351 (25 µg)) in a phase 2a trial.

## Results

### Participants

**Fig. 1:**
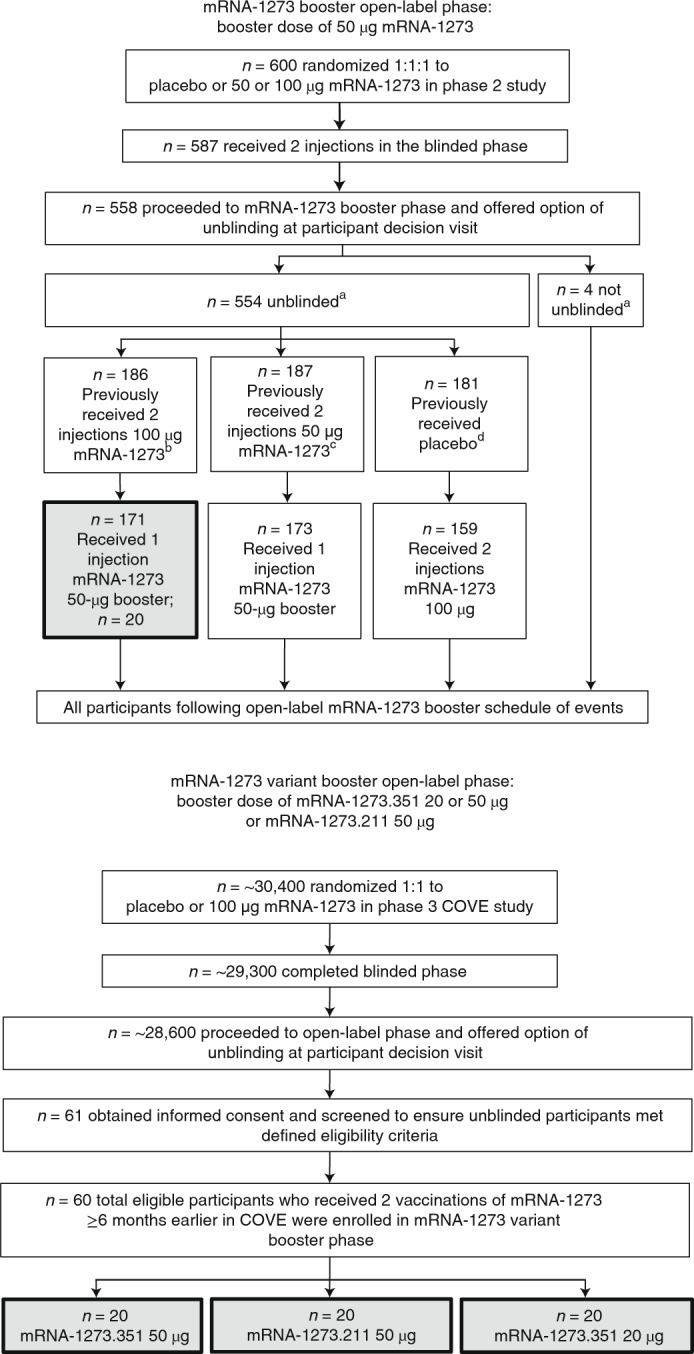
Participant flow through the mRNA-1273 booster and mRNA-1273 variant booster cohorts. In the mRNA-1273 booster phase, 20 participants who had received two injections of 100-μg mRNA-1273 completed the blinded phase and who went on to receive a single booster dose of 50-μg mRNA-1273 were selected for this preliminary analysis, with selection based on completion of day 15 visit assessments and immunogenicity sample availability. In the mRNA-1273 variant booster phase, enrollment was site specific and was based on predefined inclusion/exclusion criteria. Administration of booster doses occurred in a sequential manner (mRNA-1273 variant booster phase, which included the mRNA-1273.351 (50 µg) cohort; the mRNA-1273.211 (50 µg) cohort; and the mRNA-1273.351 (20 µg) cohort). ^a^Unblinded or not unblinded to assigned treatment in the blinded portion of the phase 2a mRNA-1273 trial. ^b^Fifteen participants declined to receive a booster. ^c^Thirteen participants declined to receive a booster. ^d^Twenty-two participants declined to receive an mRNA-1273 primary vaccination.

Among 186 participants who received two primary doses of mRNA-1273 (100 µg) in the blinded phase of the P201 study^4^ and subsequently received one booster dose of mRNA-1273 (50 µg) in the mRNA-1273 booster phase, 20 participants were randomly selected for inclusion in this interim analysis based on visit assessments completed and sample availability of pre-booster sera (Fig. 1).

Among 14,711 participants who received two primary doses of mRNA-1273 (100 µg) in the phase 3 COVE trial^5^, 60 participants were selected to enter the mRNA-1273 variant booster phase of the P201 trial and received a single booster dose of mRNA-1273.351 (50 μg; *n* = 20), mRNA-1273.211 (50 μg; *n* = 20) or mRNA-1273.351 (20 μg; *n* = 20). As one participant from the 20-μg mRNA-1273.351 group was lost to follow-up at day 29 and, thus, excluded, the analysis sample size for this cohort is 19.

The baseline demographic characteristics of the four groups of participants who received booster doses of the parent mRNA-1273 vaccine or the modified mRNA-1273 vaccines were generally similar (Table 1). Most of the participants were white and not Hispanic or Latino. The mean age of participants who received boosters of mRNA-1273 (50 µg), mRNA-1273.351 (50 µg), mRNA-1273.211 (50 µg) or mRNA-1273.351 (20 µg) was 63.8, 53.9, 55.6 and 47.5 years, respectively. The duration (mean (s.d.)) between completion of the mRNA-1273 primary series and the booster dose for mRNA-1273 (50 µg), mRNA-1273.351 (50 µg), mRNA-1273.211 (50 µg) or mRNA-1273.351 (20 µg) was 6.7 (0.5), 6.2 (0.3), 6.2 (0.4) and 6.2 (0.3) months, respectively.

**Table 1:** Demographics and clinical characteristics

	mRNA-1273 (50 µg)	mRNA-1273.351 (50 µg)	mRNA-1273.211 (50 µg)	mRNA-1273.351 (20 µg)
Characteristic *n* (%)	*n* = 20	*n* = 20	*n* = 20	*n* = 20
Mean (range) age, years	63.8 (38–76)	53.9 (27–70)	55.6 (28–79)	47.5 (26–67)
Sex				
Male	8 (40)	11 (55)	12 (60)	5 (25)
Female	12 (60)	9 (45)	8 (40)	15 (75)
Race				
White	20 (100)	19 (95)	19 (95)	20 (100)
Black or African American	0	0	0	0
Asian	0	1 (5)	0	0
American Indian or Alaska Native	0	0	1 (5)	0
Native Hawaiian or other Pacific Islander, Multiracial, Other, Not reported, Unknown	0	0	0	0
Ethnicity				
Hispanic or Latino	0	0	1 (5)	1 (5)
Not Hispanic or Latino	20 (100)	20 (100)	19 (95)	19 (95)
Not reported or Unknown	0	0	0	0
Time interval between second dose of mRNA-1273 during the primary series and the booster dose				
Mean (s.d.), months^a^	6.7 (0.5)	6.2 (0.3)	6.2 (0.4)	6.2 (0.3)
Range, months	5.9–7.5	5.6–6.6	5.4–6.8	5.5–6.6
Body mass index, kg m^−^^2^				
Mean (s.d.)	26.2 (2.1)	30.3 (6.5)^b^	33.0 (7.5)	33.3 (6.6)

^a^Calculated with 30 d per month

^b^Missing data for one participant

### Safety

**Fig. 2:**
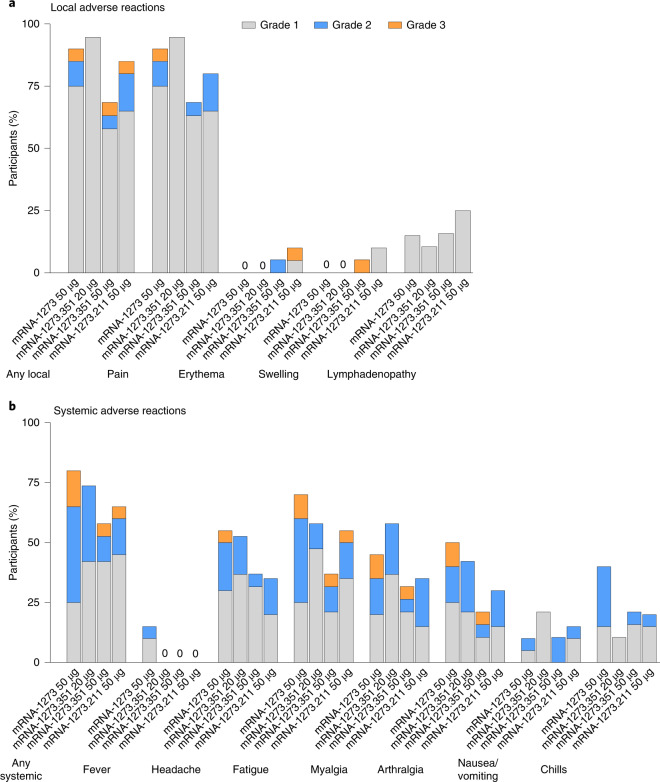
ARs within 7 d after booster dose. The percentage of participants who reported local (**a**) and systemic (**b**) ARs is shown for 20 participants who received a booster dose of mRNA-1273 (50 µg), 19 participants who received a booster dose of mRNA-1273.351 (20 µg), 19 participants who received a booster dose of mRNA-1273.351 (50 µg) and 20 participants who received a booster dose of mRNA-1273.211 (50 µg). One participant each in the mRNA-1273.351 50-µg and 20-µg groups did not report results for solicited ARs and were excluded from the analysis.

The percentages of participants with solicited local and systemic adverse reactions (ARs) were generally similar among the booster groups (Fig. 2 and Supplementary Table 1); most solicited local and systemic ARs were mild (grade 1) or moderate (grade 2). Frequencies of any grade 3 solicited local or systemic ARs after the booster doses ranged from 10% to 15%, and there were no grade 4 solicited local or systemic ARs. The most common local AR was injection site pain. The most common systemic ARs after the booster doses were fatigue, headache, arthralgia and myalgia. Fever was reported by three participants (15.0%) after the booster dose of mRNA-1273 only. No serious ARs were reported.

### Immunogenicity assessments

#### D614G and B.1.351 neutralization before and after booster

**Fig. 3:**
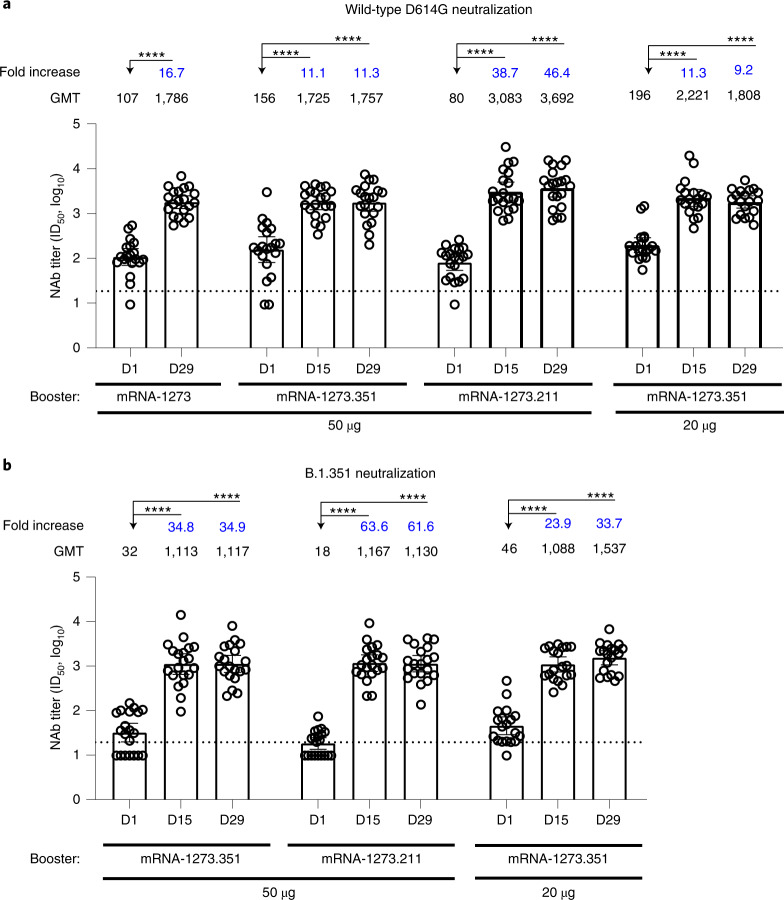
Neutralization of wild-type D614G and B.1.351 by participant serum collected immediately before and after boosters, as measured by the lentiviral-based PsVN assay. Wild-type D614G neutralization (**a**) and B.1.351 neutralization (**b**) in a validated recombinant lentivirus-based SARS-CoV-2 pseudovirus assay by serum from participants (*n* = 20 participants per booster cohort). Sera samples were collected immediately before receiving a booster (day 1) and on days 15 and 29 after the booster dose of mRNA-1273 (50 µg), mRNA-1273.351 (50 or 20 µg) or mRNA-1273.211 (50 µg). Participant sera in the mRNA-1273 (50 µg) booster group were not assessed in the B.1.351 assay. Data are presented as the geometric mean neutralizing antibody titers with 95% confidence intervals. The titers for individual participants are indicated with circles. The fold increases in titers measured at days 15 and 29 versus titers measured before the booster dose are shown. The horizonal dotted lines indicate the LLOQ. Generalized linear model was used to compare neutralization titers among groups; log_10_ titer was regressed on group, and an individual-specific random effect was included to account for individual specific variability. Two-sided *t*-test was used for post hoc group comparisons. Sidak’s method was used to adjust the *P* values for multiple comparisons. Statistical significance was determined at α < 0.01. ****P < 0.0001; ***P < 0.001; **P < 0.01; *P < 0.05. NAb, neutralizing antibody.

Wild-type D614G and B.1.351 neutralization were measured in samples collected immediately before the booster dose (day 1 (~6 months after the mRNA-1273 primary series)) and after the booster dose (day 29 in mRNA-1273 booster recipients and days 15 and 29 in mRNA-1273.351 and mRNA-1273.211 booster recipients) in a validated lentivirus PsVN assay. Sera from participants in the mRNA-1273 booster group were not assessed in the B.1.351 variant assay. The wild-type D614G virus was neutralized by most samples collected before the booster dose across all groups assessed (Fig. 3a), whereas the neutralization titers for B.1.351 were low or non-detectable before the booster dose across all groups assessed (Fig. 3b).

Neutralizing antibody titers against the wild-type D614G and B.1.351 viruses increased after each booster dose compared to day 1 titers (*P* < 0.0001 for all booster groups) (Fig. 3). Specifically, on day 29, geometric mean titers (GMTs) against the wild-type D614G virus were 16.7-, 11.3-, 46.4- and 9.2-fold higher than day 1 (pre-booster) titers in the mRNA-1273 (50 µg), mRNA-1273.351 (50 µg), mRNA-1273.211 (50 µg) and mRNA-1273.351 (20 µg) booster recipients, respectively (Fig. 3a). Similarly, on day 29, GMTs against the B.1.351 variant were 34.9-, 61.6- and 33.7-fold higher than day 1 (pre-booster) titers in the mRNA-1273.351 (50 µg), mRNA-1273.211 (50 µg) and mRNA-1273.351 (20 µg) booster recipients, respectively (Fig. 3b). These observations included participants who did not have measurable neutralizing antibodies against the wild-type D614G or B.1.351 virus before the booster dose but showed increases in their neutralizing antibody titers after the booster dose (Fig. 3); statistical analyses of this subgroup alone were not performed.

#### D614G and VOCs neutralization 1 and 6 months after primary series

An exploratory analysis of the kinetics of the immune response at 1 and 6 months after the primary mRNA-1273 vaccination series was conducted across the four groups using the VSV-based PsVN assay. This assay was previously used to evaluate the neutralizing activity of serum from participants who received mRNA-1273 in a phase 1 trial against SARS-CoV-2 wild-type virus and variants^18^.

**Fig. 4:**
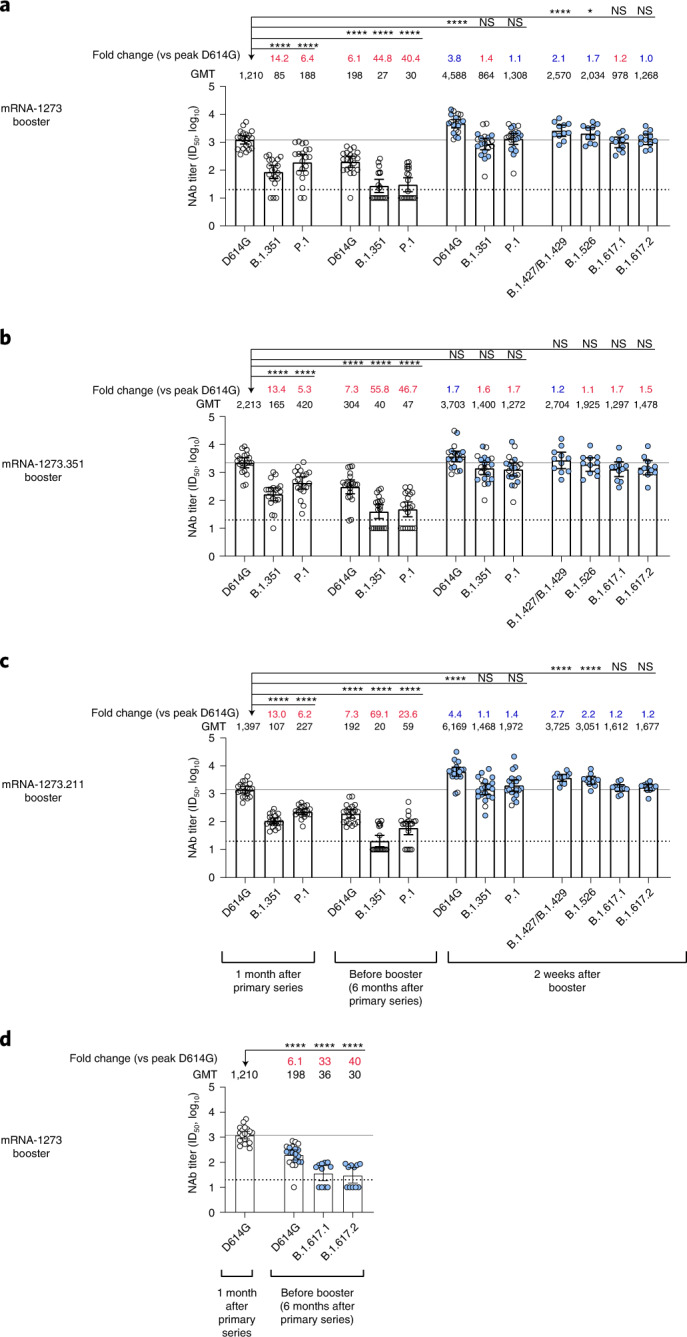
Neutralization of wild-type D614G and variants by participant serum collected 1 month after primary vaccination series and before and after boosters, as measured by the VSV-based PsVN assay. Sera samples were collected 1 month after the primary series, immediately before receiving the booster dose (6 months after the primary vaccination series) and 2 weeks after the 50-µg booster dose of mRNA-1273 (**a**), mRNA-1273.351 (**b**) or mRNA-1273.211 (**c**). *n* = 20 for wild-type D614G, B.1.351 and P.1 and *n* = 11 for B.1.427/B.1.429, B.1.526, B.1.617.1 and B.1.617.2. **d**, Neutralization of wild-type D614G 1 month after the primary series and neutralization of wild-type D614G, B.1.617.1 and B.1.617.2 immediately before the booster dose from sera samples collected from a subset of participants in the mRNA-1273 booster group (D614G, *n* = 20; B.1.617.1, *n* = 11; B.1.617.2, *n* = 11). In **a**–**d**, the GMTs against the wild-type D614G and variants measured in participants before the booster dose or 2 weeks after the booster dose were evaluated versus peak titers measured against the wild-type D614G 1 month after the primary vaccination series. Data are presented as the geometric mean neutralizing antibody titers with 95% confidence intervals. The GMT fold change versus the peak titers against the wild-type D614G virus after the primary vaccination series are shown, with red indicating fold drop and blue indicating fold rise. Results from individual participants are represented as dots on each figure. For all panels, blue colored dots indicate participants whose samples were tested (*n* = 11), and white dots indicate the remaining participants whose samples were not tested (*n* = 9). The horizonal dotted line indicates the LLOQ; the solid gray line indicates the within-cohort GMT benchmark. Generalized linear model was used to compare neutralization titers among groups; log_10_ titer was regressed on group, and an individual-specific random effect was included to account for individual specific variability. Two-sided *t*-test was used for post hoc group comparisons. Sidak’s method was used to adjust the *P* values for multiple comparisons. Statistical significance was determined at α < 0.01. ****P < 0.0001; ***P < 0.001; **P < 0.01; *P < 0.05. NAb, neutralizing antibody; NS, not significant.

One month after the primary series, wild-type D614G neutralizing antibody GMT ranged from 1,210 to 2,213 across participants in the 50-µg booster groups (Fig. 4) and was 2,758 in the mRNA-1273.351 20-µg booster group (Supplementary Fig. 1). B.1.351 and P.1 neutralizing antibody GMTs were 13- to 14-fold lower and 5- to 6-fold lower, respectively, compared to wild-type D614G at the same time point in the 50-µg groups.

Simultaneous analysis of samples using the VSV PsVN assay showed that, approximately 6 months after the mRNA-1273 primary vaccination series, neutralizing antibody levels decreased (*P* < 0.0001) compared to peak titers against wild-type D614G measured 1 month after the primary series (GMTs against wild-type D614G were 6- to 7-fold lower, and GMTs against B.1.351 and P.1 were 24- to 69-fold lower) (Fig. 4a–c). Neutralizing antibody levels against B.1.351 and P.1 were below the lower limit of quantification (LLOQ) of the assay in ~44% and 30% of samples, respectively. Neutralization of the B.1.617.2 variant was also reduced (*P* < 0.0001) 6 months after the completion of the primary series (Fig. 4d). Sera from a random subset of the 20 participants in the mRNA-1273 booster group was used to assess neutralization of B.1.617.1 and B.1.617.2 (*n* = 11 for both) 6 months after the primary series and showed a 33- to 40-fold reduction in neutralizing antibody titers against B.1.617.1 and B.1.617.2 in comparison to peak titers measured against wild-type D614G 1 month after the primary series (full mRNA-1273 interim analysis cohort; *n* = 20). Neutralizing antibody titers against B.1.617.2 fell below the LLOQ of the assay in five of 11 samples.

#### D614G and VOCs neutralization after booster

Neutralizing antibody titers against the wild-type D614G virus were measured with the VSV-based PsVN assay using samples collected 2 weeks after the booster dose and were compared against wild-type D614G GMT benchmarks from samples collected 1 month after the primary series vaccination in each group. These benchmarks were used to determine whether the boosters reached the same neutralization level shown in the pivotal study where efficacy was demonstrated (that is, levels seen for wild-type D614G where 94% efficacy was measured)^5^. Using sera collected 1 month after the mRNA-1273 (100 µg) primary series, a GMT of 1,210 in the mRNA-1273 group (50 µg), 2,213 in the mRNA-1273.351 (50 µg), 1,397 in the mRNA-1273.211 (50 µg) and 2,758 in the mRNA-1273.351 (20 µg) groups were measured (Fig. 4a–c and Supplementary Fig. 1). Compared to the wild-type D614G benchmarks for each group, the booster vaccines yielded superior (mRNA-1273 and mRNA-1273.211) or equivalent (mRNA-1273.351) GMTs against the wild-type D614G virus. Wild-type D614G neutralization was 3.8-fold (*P* < 0.0001), 1.7-fold (*P* value not significant (NS)) and 4.4-fold (*P* < 0.0001) higher 2 weeks after 50-µg booster doses of mRNA-1273, mRNA-1273.351 and mRNA-1273.211, respectively, compared to peak titers against wild-type D614G measured 1 month after the primary series (Fig. 4a–c). All three boosters, including mRNA-1273, increased neutralization against VOCs or VOIs to levels that were statistically equivalent to the wild-type D614G benchmarks, with superior titers measured versus some VOIs. Of the three booster vaccines assessed, the multivalent mRNA-1273.211 had the greatest increase in GMTs against all VOCs. Neutralization titers against B.1.351, P.1, B.1.427/B.1.429, B.1.526, B.1.617.1 and B.1.617.2 were 1.1-fold (*P* value NS), 1.4-fold (*P* value NS), 2.7-fold (*P* < 0.0001), 2.2-fold (*P* < 0.0001), 1.2-fold (*P* value NS) and 1.2-fold (*P* value NS) higher, respectively, 2 weeks after the mRNA-1273.211 booster compared to peak titers against the wild-type D614G measured 1 month after the primary series (Fig. 4c).

Importantly, neutralizing antibody titers against wild-type D614G and B.1.351 measured using the clinically validated lentivirus and research grade VSV-based PsVN assays were highly correlated (*r* = 0.9161 against wild-type D614G and *r* = 0.9435 against B.1.351; Supplementary Fig. 2).

## Discussion

This preliminary evaluation describes the antibody persistence of mRNA-1273 and the safety and immunogenicity of a booster dose of mRNA-1273, mRNA-1273.351 or mRNA-1273.211 in a subset of 80 participants who had been vaccinated ~6 months previously with the authorized dose and schedule of mRNA-1273. Antibody titers against the wild-type D614G peaked 1 month after completion of the primary series and subsequently declined over the 5 months before the booster dose^18^. These results are consistent with those reported in a study using a lentiviral PsVN assay, in which monitoring of neutralizing antibody levels was performed up to 6 months after completion of the mRNA-1273 primary series^19^. Reduction of neutralizing antibody titers against B.1.351 and P.1 was evident 1 month after the primary series to a greater degree than that observed 7 d after the primary series^18^, likely due to further affinity maturation of B cells and alteration of the available antibody repertoire. Additional reduction or complete loss of detectible levels of neutralizing antibody ~6 months after the primary series was evident against B.1.351, P.1 and B.1.617.2.

The safety profiles after single booster injections of mRNA-1273 (50 µg), mRNA-1273.351 (20 or 50 µg) and mRNA-1273.211 (50 µg) were generally similar to those observed after the mRNA-1273 primary series in the previously reported phase 2 and 3 studies^12,14^. The most common systemic ARs after the booster doses were fatigue, headache, arthralgia and myalgia, which occurred at similar-to-lower frequencies for the boosters than after receipt of the mRNA-1273 (100 μg) primary series.

Booster vaccination with mRNA-1273, mRNA-1273.351 and mRNA-1273.211 induced strong anamnestic responses, indicative of a robust B cell memory response^13^. Neutralizing antibody titers against the wild-type D614G virus after a booster dose were up to 4.4-fold higher than peak titers after the primary series. Neutralizing antibody titers against several VOCs (that is, B.1.351, P.1 and B.1.617.2) increased after the booster dose, with titers against several variants approaching or exceeding those measured after the primary series against the wild-type D614G virus (Fig. 4). Increased titers against the VOCs suggest that further maturation of antibodies is feasible after a two-dose primary series of mRNA-1273, regardless of the composition of the booster dose. Furthermore, boosting with mRNA-1273.351 and mRNA-1273.211 appeared to produce numerically greater neutralizing antibody titers against the B.1.351 variant than with mRNA-1273, although formal conclusions regarding the significance of these differences cannot be made.

The multivalent mRNA-1273.211 (50 µg) booster yielded a GMT ratio rise ≥1 against all VOCs and VOIs 2 weeks after the booster dose versus peak wild-type D614G titers measured 1 month after the primary series vaccination (Fig. 4c). This rise was significant for B.1.427/B.1.429 and B.1.526, indicating that variant neutralization GMTs after the booster were higher than peak wild-type D614G virus GMTs after the primary series in the samples from this cohort, potentially increasing breadth of coverage against VOCs or VOIs.

There are some limitations related to this preliminary analysis. First, the results presented here are based on treatment groups that were not randomized. Instead, participants were assigned sequentially, given the different time frames of availability of the new vaccine formulations. The sample size was small (*n* = 20 per group) to facilitate rapid initiation of additional studies, which are needed to support the safe and effective use of a COVID-19 booster vaccine and to inform ongoing vaccination preparedness strategies regarding the need for booster doses as the pandemic evolves. Participants in this interim analysis were predominantly white and non-Hispanic or Latino (95–100% across booster groups), limiting generalizability to other races and ethnicities. Sex distributions and mean body mass index and age were not equivalent across the four booster groups; however, these differences are expected given the small cohort sizes. Although the lentiviral-based PsVN assay used in this evaluation is validated, the VSV-based PsVN assay used in the evaluation of samples against variants is a research grade assay that has not been validated. Nevertheless, the high correlation between the clinically validated lentivirus-based PsVN assay and research grade VSV-based PsVN assay provides support for the utility of the latter in improving the efficiency and time needed to perform such clinical analyses. Moreover, although these data are encouraging, in the absence of a correlate of protection it cannot be definitively determined whether the neutralization titers elicited by the mRNA-1273, mRNA-1273.351 and mRNA-1273.211 booster doses are protective against the B.1.351, P.1 or B.1.617.2 variants. Finally, because the participants in this study were originally enrolled in two different clinical trials, comparison of the results from mRNA-1273.211 and mRNA-1273.351 boosting with those of mRNA-1273 should be interpreted with caution.

The emergence of SARS-CoV-2 variants and the ability of the virus to partially overcome natural or vaccine-induced immunity has served as a call to action. Although a correlate of protection has not been established for SARS-CoV-2 infection or COVID-19 disease, lack of detectable neutralization against VOCs after ~6 months in some participants might be indicative of waning protection. However, it should be noted that an anamnestic response upon viral exposure is likely based on the induction of immune memory from the booster dose. The mRNA platform approach against SARS-CoV-2 VOCs in this trial appears to be effective in developing wild-type and variant-specific booster vaccines, with boosters increasing neutralizing titers against the wild-type D614G virus and against key VOCs and VOIs. Of note, significantly higher neutralizing titers against wild-type D614G, B.1.427/B.1.429 and B.1.526 and statistically equivalent titers against B.1.351, P.1 and B.1.617.2 were measured 2 weeks after the booster dose compared to wild-type D614G neutralization measured 1 month after the primary series in participants who received the multivalent mRNA-1273.211 booster. Further research is needed to determine the clinical significance of these preliminary results. Although this trial evaluated the performance of booster vaccines that encode the original strain or the B.1.351 S protein, this strategy could be employed in the future to vaccinate against new VOCs through the development of new variant-specific vaccines.

## Methods

### Study design

This ongoing phase 2a mRNA-1273 trial (protocol mRNA-1273-P201; NCT04405076, hereafter referred to as P201) is being conducted at eight sites in the United States and consists of three parts: a blinded phase where participants received two dose levels (50 or 100 µg) of mRNA-1273 primary series, which was previously published^4^, and two open-label intervention phases where participants who had previously received two primary doses of mRNA-1273 (100-µg dose group only) received either a booster dose of mRNA-1273 (50 µg; referred to herein as the ‘mRNA-1273 booster phase’) or mRNA-1273.351 (50 µg), mRNA-1273.211 (50 µg) or mRNA-1273.351 (20 µg; collectively, the three groups are referred to herein as the arms within the ‘mRNA-1273 variant booster phase’) (Fig. 1). Participants were enrolled sequentially into each booster arm. The objectives of these two open-label intervention phases were to assess the safety and immunogenicity of booster doses of mRNA-1273, mRNA-1273.211 and mRNA-1273.351. Additional details pertaining to study design are included in the Supplementary Methods.

All study materials, including the protocol, amendments and informed consent, were approved by a central institutional review board (Advarra). All participants provided written informed consent before enrollment and participation in study procedures. A safety monitoring committee (SMC), composed of external experts, reviewed data at prespecified time points during the blinded phase of the study. For the open-label phases, the SMC was informed of protocol amendments but met only on an ad hoc basis if any safety concerns arose; there were no significant safety concerns to trigger an ad hoc meeting of the SMC.

### Participants

Eligible participants were healthy adults ≥18 years of age at the time of consent. To be eligible for inclusion into the mRNA-1273 booster phase, participants must have been previously enrolled in the blinded portion of the mRNA-1273 P201 study (NCT04405076) and received two doses of mRNA-1273 (50 or 100 µg). Only participants who received 100-µg doses of mRNA-1273 in the P201 study were included in this analysis. To be eligible for inclusion into the mRNA-1273 variant booster phase, participants must have been enrolled in the mRNA-1273-P301 COVE (NCT04470427) study and received two doses of mRNA-1273, with their second dose ≥6 months before enrollment in P201. Participants were enrolled in the blinded phase of the trial from 29 May 2020 to 8 July 2020. In the open-label phases, participants were enrolled from 28 January 2021 to 2 April 2021 in the mRNA-1273 booster phase (*n* = 345 enrolled) and from 10 March 2021 to 19 March 2021 in the mRNA-1273 variant booster phase (*n* = 60 enrolled). Study enrollment is complete, and the mRNA-1273 booster phase is expected to be completed in October 2021. Additional inclusion and exclusion criteria are provided in the protocol, which is included with the supplementary materials.

### Vaccines

mRNA-1273 encodes the S protein of the Wuhan-Hu-1 isolate of SARS-CoV-2, whereas mRNA-1273.351 encodes the S protein of the SARS-CoV-2 B.1.351 variant; both vaccines include two proline mutations introduced to stabilize the S protein into the prefusion conformation (Supplementary Table 2). mRNA-1273.211 was a 1:1 mix of mRNA-1273 (25 µg) and mRNA-1273.351 (25 µg), for a total dose of 50 µg. All vaccines were formulated in lipid nanoparticles as previously described^5^.

### Safety assessments

Participants completed an electronic diary for 7 d after receiving the booster dose to record solicited systemic ARs, local ARs (including injection site erythema and swelling/induration) and daily oral body temperatures. Trained site personnel called participants to assess safety every 4 weeks for 6 months after the last dose.

### Immunogenicity assessments

Blood was collected 1 month after the primary vaccination series, immediately before the booster dose (day 1) and at days 8, 15, 29, 57 and 181 after the booster dose. In this interim analysis, neutralizing antibody titers of sera collected 1 month after the primary series, immediately before the booster dose and at days 15 and 29 after the booster dose are reported. A validated lentivirus PsVN assay (described below) was used to analyze samples collected immediately before the booster dose (day 1) and at days 15 and 29 after the booster. Additionally, a research grade recombinant VSV-based pseudovirus assay (described below) was used to assess neutralizing antibody titers against a panel of SARS-CoV-2 variants from sera collected 1 month after the primary series, immediately before the booster dose and day 15 after the booster dose. This assay has previously been used to evaluate neutralization against a panel of variants from sera collected 7 d after the primary series^18^.

### Recombinant lentiviral-based PsVN assay (validated clinical assay)

SARS-CoV-2 neutralizing antibodies were quantified using lentivirus particles that incorporate SARS-CoV-2 S protein (Wuhan-Hu-1 isolate mutated to contain D614G) or the B.1.351 variant S protein (L18F-D80A-D215G-∆L242-∆A243-∆L244-K417N-E484K-N501Y-D614G-A701V) on their surface and express firefly luciferase reporter gene for quantitative measurements of infection by relative luminescence units (RLUs) as described^20^. The virus is applied to stably transduced 293T cells expressing high levels of angiotensin-converting enzyme 2 (ACE2) (293T/ACE2 cells), with or without pre-incubation with antibodies (control antibodies or serum samples); the presence of neutralizing antibodies reduces infection and results in lower RLUs. Serial dilution of antibodies or serum samples can be used to produce a dose–response curve. Neutralization is measured as the serum dilution at which the RLUs are reduced by 50% (50% inhibitory dilution (ID_50_)) relative to the mean RLUs in virus control wells (cells + virus but no control antibody or sample) after subtraction of the mean RLUs in cell control wells (cells only).

### Recombinant VSV-based PsVN assay (research grade assay)

To perform the recombinant VSV-based PsVN assay, codon-optimized full-length S protein of the D614G and variant sequences (Supplementary Table 2) were cloned into a pCAGGS vector. To make SARS-CoV-2 full-length S pseudotyped recombinant VSV-ΔG-firefly luciferase virus, BHK-21/WI-2 cells (Kerafast, EH1011) were transfected with the S expression plasmid and subsequently infected with VSVΔG-firefly-luciferase as previously described^21^. For the neutralization assay, serially diluted serum samples were mixed with pseudovirus and incubated at 37 °C for 45 min. The virus/serum mix was subsequently used to infect A549-hACE2-TMPRSS2 cells for 18 h at 37 °C before adding ONE-Glo reagent (Promega, E6120) for measurement of luciferase signal (RLU). The percentage of neutralization was calculated based on RLUs of the virus-only control and subsequently analyzed using 4-parameter logistic curve (Prism 8). Neutralization curves of SARS-CoV-2-negative human serum from two representative runs are presented in Supplementary Fig. 3.

### Statistical analysis

No hypothesis testing and no formal power calculations were performed. This was an exploratory interim analysis, with the formal protocol-prespecified interim analysis pending the completion of the trial. This exploratory interim analysis was prepared to enable sharing of key neutralization results in light of the ongoing discussions around booster vaccines due to concerns with waning vaccine-induced immunity that is more pronounced with key VOCs. Descriptive summary statistics of the safety endpoints are provided; statistical tests were not performed for comparisons of these endpoints. Generalized linear model was used to compare neutralization titers between groups; log_10_ titer was regressed on group, and an individual-specific random effect was included to account for individual-specific variability. Two-sided *t*-test was used for post hoc group comparisons. Sidak’s method was used to adjust the *P* values for multiple comparisons. Statistical significance was determined at α < 0.01. GMTs and geometric mean fold rises (GMFRs) were calculated based on log-transformed titers, and two-sided 95% confidence intervals were based on the *t-*distribution of the log-transformed titers or the difference in the log-transformed titers for GMT and GMFR, respectively, and were then back-transformed to the original scale.

Analysis of the study participant sera collected 1 month after the primary series was used to establish the GMT benchmarks for each group that were further used to derive GMT ratios after the booster dose. Spearman non-parametric correlation was used for assay correlation.

### Reporting Summary

Further information on research design is available in the Nature Research Reporting Summary linked to this article.

## Online content

Any methods, additional references, Nature Research reporting summaries, source data, extended data, supplementary information, acknowledgements, peer review information; details of author contributions and competing interests; and statements of data and code availability are available at 10.1038/s41591-021-01527-y.

## Supplementary information

Supplementary Information Supplementary Figs. 1–3, Supplementary Tables 1 and 2, List of Site Investigators, Supplementary Methods and Protocol

Reporting Summary

## Data Availability

The data supporting the findings of this study are available in this article and its Supplementary Information.
