# Supplementary material for: Safety and immunogenicity of SARS-CoV-2 variant mRNA vaccine boosters in healthy adults: an interim analysis
Source: Nat Med. 2021 Sep 15;27(11):2025–31. doi: 10.1038/s41591-021-01527-y (PMC8604720; doi:10.1038/s41591-021-01527-y)
Supplement: Supplementary file 2 — Reporting Summary [file 41591_2021_1527_MOESM2_ESM.pdf]

nature portfolio

Corresponding author(s): Darin K. Edwards

Last updated by author(s): Aug 31, 2021

Reporting Summary

Nature Portfolio wishes to improve the reproducibility of the work that we publish. This form provides structure for consistency and transparency in reporting. For further information on Nature Portfolio policies, see our Editorial Policies and the Editorial Policy Checklist.

Statistics

For all statistical analyses, confirm that the following items are present in the figure legend, table legend, main text, or Methods section.

n/a	Confirmed	
☐	☒	The exact sample size (n) for each experimental group/condition, given as a discrete number and unit of measurement
☐	☒	A statement on whether measurements were taken from distinct samples or whether the same sample was measured repeatedly
☐	☒	The statistical test(s) used AND whether they are one- or two-sided<br>*Only common tests should be described solely by name; describe more complex techniques in the Methods section.*
☒	☐	A description of all covariates tested
☒	☐	A description of any assumptions or corrections, such as tests of normality and adjustment for multiple comparisons
☐	☒	A full description of the statistical parameters including central tendency (e.g. means) or other basic estimates (e.g. regression coefficient) AND variation (e.g. standard deviation) or associated estimates of uncertainty (e.g. confidence intervals)
☐	☒	For null hypothesis testing, the test statistic (e.g. F, t, r) with confidence intervals, effect sizes, degrees of freedom and P value noted<br>*Give P values as exact values whenever suitable.*
☒	☐	For Bayesian analysis, information on the choice of priors and Markov chain Monte Carlo settings
☒	☐	For hierarchical and complex designs, identification of the appropriate level for tests and full reporting of outcomes
☒	☐	Estimates of effect sizes (e.g. Cohen's d, Pearson's r), indicating how they were calculated

Our web collection on statistics for biologists contains articles on many of the points above.

Software and code

Policy information about availability of computer code

Data collection	No software used for data collection.
Data analysis	Prism (v.8; GraphPad Software, Inc.) for figure development; SAS for statistical analysis (v. 9.4 or above)

For manuscripts utilizing custom algorithms or software that are central to the research but not yet described in published literature, software must be made available to editors and reviewers. We strongly encourage code deposition in a community repository (e.g. GitHub). See the Nature Portfolio guidelines for submitting code & software for further information.

Data

Policy information about availability of data

All manuscripts must include a data availability statement. This statement should provide the following information, where applicable:

- Accession codes, unique identifiers, or web links for publicly available datasets
- A description of any restrictions on data availability
- For clinical datasets or third party data, please ensure that the statement adheres to our policy

The authors declare that the data supporting the findings of this study are available within this Article and it's Supplementary Information.

Field-specific reporting

Please select the one below that is the best fit for your research. If you are not sure, read the appropriate sections before making your selection.

☒ Life sciences ☐ Behavioural & social sciences ☐ Ecological, evolutionary & environmental sciences

For a reference copy of the document with all sections, see nature.com/documents/nr-reporting-summary-flat.pdf

Life sciences study design

All studies must disclose on these points even when the disclosure is negative.

Sample size	There was no formal hypothesis testing in this study. The number of proposed participants was considered sufficient to provide a descriptive summary of the safety and immunogenicity of different dose levels of mRNA-1273. For this interim analysis, we randomly selected n=20 subject samples from the Part B booster group and a total of n=60 from Part C.
Data exclusions	20 participants in Part B were randomly selected for this analysis. All 60 Part C participants were included in this analysis (n=20 each cohort). Data from those 80 participants were included in this paper. In the mRNA-1273.351 20 ug booster group, 1 participant was lost to follow up. There were otherwise no data exclusions.
Replication	Data was collected once for each trial participant at the indicated time points.
Randomization	During Part A, the blinded phase of the study, within each age cohort, approximately 300 participants were randomly assigned in 1:1:1 ratio to receive mRNA-1273 50 µg, mRNA-1273 100 µg, or placebo. The randomization was blinded using a centralized Interactive Response Technology (IRT), in accordance with pregenerated randomization schedules.<br><br>Parts B and C of the study were open label. In Part B, participants who received placebo in Part A of the study had the option to receive 2 injections of open-label mRNA-1273. Participants who received 1 or 2 doses of 50 µg or 100 µg mRNA-1273 in Part A were offered a single booster dose of mRNA-1273 (50 µg) in Part B. 20 of the participants who had originally received 2 priming doses of 100 µg mRNA-1273 and received a single booster dose of 50 µg mRNA-1273 were randomly selected based on visit assessments completed and sample availability for this analysis. In Part C, enrollment and vaccination in each study arm was sequential. 60 participants who had originally received 2 priming dose of 100 ug mRNA-1273 in the COVE study were enrolled in Part C. The first 20 participants were enrolled and dosed on open-label day 1 with the mRNA-1273.351 50 µg dose. Upon completion of the first 50 µg dose arm, 20 participants were enrolled in the second arm and received the mRNA-1273/mRNA-1273.351 mixture (50 µg total dose). Following completion of the second arm, 20 participants were enrolled and dosed with the mRNA-1273.351 20 µg dose.
Blinding	Part A of was observer blind. The investigator, study staff, study participants, site monitors, and sponsor personnel (or its designees) were blinded to the investigational product administered until study end or initiation of Part B, with the following exceptions: unblinded pharmacy personnel, unblinded site monitors, and a limited number of unblinded sponsor and clinical research organization personnel who performed the primary study analysis and prepared the Clinical Study Report. Parts B and C of the study were open label. However, the lab personnel who were involved in the sample testing were blinded at the time of sample testing.

Reporting for specific materials, systems and methods

We require information from authors about some types of materials, experimental systems and methods used in many studies. Here, indicate whether each material, system or method listed is relevant to your study. If you are not sure if a list item applies to your research, read the appropriate section before selecting a response.

Materials & experimental systems

n/a	Involved in the study	
☒	☐	Antibodies
☐	☒	Eukaryotic cell lines
☒	☐	Palaeontology and archaeology
☒	☐	Animals and other organisms
☐	☒	Human research participants
☐	☒	Clinical data
☒	☐	Dual use research of concern

Methods

n/a	Involved in the study	
☒	☐	ChIP-seq
☒	☐	Flow cytometry
☒	☐	MRI-based neuroimaging

Eukaryotic cell lines

Policy information about cell lines

Cell line source(s)	There are 4 cell lines used in this manuscript. A549 cells were purchased from ATCC (Cat# CCL-185) and engineered to express ACE2 and TMPRSS2 as described previously (https://www.nature.com/articles/s41586-020-2622-0) to make A549-ACE2-TMPRSS2. 293T/17 [HEK 293T/17] cells were obtained from ATCC (cat. # CRL-11268). 293T/ACE2 (also called 293T-

	hACE2.MF) were provided by Drs. Mike Farzan and Huihui Mu at Scripps. BHK-21/WI-2 cells (Kerafast cat. # EH1011) were provided by Dr. Michael Whitt at The University of Tennessee Health Science Center.
Authentication	A549-ACE2-TMPRSS2 is not authenticated in terms of karyotyping; however, expression of both ACE2 and TMPRSS2 has been confirmed. 293T/ACE2 cells were not formally authenticated and were characterized by flow cytometry for ACE2 expression levels. BHK-21/WI-2 cells have been characterized by isoenzyme analysis which confirmed that the cells are from Syrian golden hamsters.
Mycoplasma contamination	A549-ACE2-TMPRSS2 cells are tested biannually and have been negative for mycoplasma. The 293T/17, 293T/ACE2, and BHK-21/WI-2 cell lines were routinely tested for mycoplasma and tested negative during the course of these experiments.
Commonly misidentified lines (See ICLAC register)	None

Human research participants

Policy information about studies involving human research participants

Population characteristics	Participants were males and females 18 years of age or older, were in good health according to the assessment of the investigator, and could comply with study procedures. Negative pregnancy tests were required at Screening and before vaccine administration for female participants of childbearing potential.
Recruitment	The blinded portion of the P201 clinical trial recruited healthy volunteer participants from the general population across 8 clinical study sites throughout the United States. Following Moderna's emergency use authorization in December 2020, the P201 study protocol was amended to allow participants from the study to be unblinded during a Participant Decision Visit, and move to Part B open-label part of the study.<br><br>In Part B, participants who were originally randomized to placebo were offered to receive 2 doses of mRNA-1273 100ug, participants who were originally randomized to mRNA-1273 (50 or 100ug) were offered to receive a single booster dose of mRNA-1273 50ug. Of the participants who consented to move to Part B of the study and receive a single booster dose of mRNA-1273, n=20 were randomly selected for this preliminary analysis, based on completion of their day 15 visit assessments and respective samples availability for analysis. In Part B, potential bias was minimized by the random selection of the 20 participants.<br><br>In Part C of the study, n=60 subjects at a single study site participants from Moderna's Phase 3 (P301 COVE) study who had previously received 2 doses of mRNA-1273, with the 2nd dose at least 6 months prior to start of Part C were offered to roll over into P201 Part C upon consent and meeting Part C specified I/E criteria as defined in the study protocol. Upon consent, Part C subjects were dosed with a single booster of 1 of the 3 variant vaccines.
Ethics oversight	Central IRB (Advarra)<br><br>All participants provided written informed consent prior to enrollment and participation in study procedures. The IRB reviewed each individual sites' participant compensation.

Note that full information on the approval of the study protocol must also be provided in the manuscript.

Clinical data

Policy information about clinical studies
All manuscripts should comply with the ICMJE guidelines for publication of clinical research and a completed CONSORT checklist must be included with all submissions.

Clinical trial registration	NCTC04405076
Study protocol	Protocol provided with the manuscript submission
Data collection	Data were collected at 8 US sites.<br><br>Investigator sites:<br>Benchmark Research, 3100 Red River St, Suite 1, Austin, TX 78705;<br>Meridian Clinical Research, 340 Eisenhower Drive, Suite 1200, Savannah, GA 31406;<br>Meridian Clinical Research, 1410 North 13th Street, Suite 5, Norfolk, NE 68701;<br>Meridian Clinical Research, 4802 Sunnybrook Dr., Sioux City, IA 51106;<br>Alliance for Multispecialty Research, 700 Medical Drive, Suite 110, Newton, Kansas 67114;<br>Trial Management Associates, 3806 Peachtree Avenue, Suite 200, Wilmington, NC 28403;<br>Alliance for Multispecialty Research, LLC, 1010 Carondelet Drive, Suite 426,Kansas City, MO 64114;<br>Benchmark Research – San Angelo, 3605 Executive Drive, San Angelo, TX 76904<br><br>Study time periods:<br>Part A: first subject dosed – 29May2020 and last subject final dose – 8Jul2020<br>Part C: first subject dosed – 10Mar2021 and last subject last date – 19Mar2021

Outcomes

The primary and secondary objectives of Part B were:
-To evaluate the safety and reactogenicity of 50 µg of mRNA-1273 vaccine administered as a single booster dose or 100 µg of mRNA-1273 administered as 2 doses 28 days apart
-To evaluate the immunogenicity of 50 µg of mRNA-1273 vaccine administered as a single booster dose or 100 µg of mRNA-1273 administered as 2 doses 28 days apart, as assessed by the level of specific bAb
-To evaluate the immunogenicity of 50 µg of mRNA-1273 vaccine administered as a single booster dose or 100 µg of mRNA-1273 administered as 2 doses 28 days apart, as assessed by the titer of nAb

The primary and secondary objectives of Part C were:
-To evaluate the safety and reactogenicity of 2 dose levels (20 and 50 µg) of mRNA-1273.351 or 1 dose level of a mixture of 25 µg mRNA-1273 and 25 µg mRNA-1273.351 (mRNA-1273/mRNA-1273.351 mixture) (50 µg total) vaccine administered as a single booster dose after completing a 2-dose vaccination schedule of mRNA-1273 at least 6 months earlier
-To evaluate the immunogenicity of 2 dose levels (20 and 50 µg) of mRNA-1273.351 or 1 dose level of mRNA-1273/mRNA-1273.351 mixture (50 µg total) vaccine administered as a single booster dose after completing a 2-dose vaccination schedule of mRNA-1273 at least 6 months earlier, as assessed by the level of specific nAb directed against the S2P antigen derived from B.1.351 variant
-To evaluate the immunogenicity of 2 dose levels (20 and 50 µg) of mRNA-1273.351 or 1 dose level of mRNA-1273/mRNA-1273.351 mixture (50 µg total) vaccine administered as a single booster dose after completing a 2-dose vaccination schedule of mRNA-1273 at least 6 months earlier, as assessed by the level of specific bAb directed against the S2P antigen derived from B.1.351 variant

In both Part B and Part C, the primary outcomes were pre-defined as related to safety and reactogenicity as the main goal of the study was to establish an acceptable safety profile of the vaccine administered at the dose levels described and according to the schedule outlined. Similarly, secondary outcomes were pre-defined as related to immunogenicity to investigate the immune response, as measured by both binding antibodies and neutralizing antibodies, following administration of a single 50 mg booster dose of mRNA-1273 or 2 doses of 100 mg each of mRNA1273 in Part B. In Part C, the immune response was investigated by pre-defined outcomes examining 2 dose levels (20 and 50 µg) of mRNA-1273.351 or 1 dose level of mRNA-1273/mRNA-1273.351 mixture (50 µg total) vaccine administered as a single booster dose after completing a 2-dose vaccination schedule of mRNA-1273 at least 6 months earlier.
